# Pan-Cancer Single-Cell Analysis Reveals the Core Factors and Pathway in Specific Cancer Stem Cells of Upper Gastrointestinal Cancer

**DOI:** 10.3389/fbioe.2022.849798

**Published:** 2022-05-13

**Authors:** Leijie Li, Yujia Zhang, Yongyong Ren, Zhiwei Cheng, Yuening Zhang, Xinbo Wang, Hongyu Zhao, Hui Lu

**Affiliations:** ^1^ SJTU-Yale Joint Center for Biostatistics and Data Science, School of Life Sciences and Biotechnology, Shanghai Jiao Tong University, Shanghai, China; ^2^ Department of Biostatistics, Yale University, New Haven, CT, United States

**Keywords:** upper gastrointestinal cancers, cancer stem cell, single-cell sequence data, pan-cancer analysis, oncogene

## Abstract

Upper gastrointestinal cancer (UGIC) is an aggressive carcinoma with increasing incidence and poor outcomes worldwide. Here, we collected 39,057 cells, and they were annotated into nine cell types. By clustering cancer stem cells (CSCs), we discovered the ubiquitous existence of sub-cluster CSCs in all UGICs, which is named upper gastrointestinal cancer stem cells (UGCSCs). The identification of UGCSC function is coincident with the carcinogen of UGICs. We compared the UGCSC expression profile with 215,291 single cells from six other cancers and discovered that UGCSCs are specific tumor stem cells in UGIC. Exploration of the expression network indicated that inflammatory genes (*CXCL8*, *CXCL3*, *PIGR*, and *RNASE1*) and Wnt pathway genes (*GAST*, *REG1A*, *TFF3*, and *ZG16B*) are upregulated in tumor stem cells of UGICs. These results suggest a new mechanism for carcinogenesis in UGIC: mucosa damage and repair caused by poor eating habits lead to chronic inflammation, and the persistent chronic inflammation triggers the Wnt pathway; ultimately, this process induces UGICs. These findings establish the core signal pathway that connects poor eating habits and UGIC. Our system provides deeper insights into UGIC carcinogens and a platform to promote gastrointestinal cancer diagnosis and therapy.

## Introduction

Upper gastrointestinal cancer (UGIC), including head and neck squamous cell carcinoma (HNSCC), esophageal cancer (EC), and gastric cancer (GC), is one of the malignant tumors that seriously threaten the human health (Yamada et al., 2011). Its occurrence is mainly associated with unhealthy eating habits and lifestyle and their consequences, including low intake of fruits and vegetables (Akhtar, 2013), smoking (Gandini et al., 2008), drinking (Goldstein et al., 2010; Zhang et al., 2012; González et al., 2013), and high body mass index (BMI). The global incidence of UGIC has significantly increased in recent years (Bray et al., 2018). Patients with UGIC account for a large proportion of all patients with malignant tumors (Sung et al., 2021). UGIC has a poorer prognosis and lower overall survival rate than other cancers (Sung et al., 2021). GC is the fifth most prevalent cancer and the third leading death cause of patients with cancers on a global scale (Yin et al., 2020). The 5-year survival rate of patients with EC is not more than 20% worldwide (Zhang, 2013). Because of the increasing incidence, the high relapse and metastasis rate, and the low overall survival rate, studies on the molecular mechanism of UGICs or gastrointestinal pan-cancer are imperative.

In recent years, the growing number of patients has prompted many studies on gastrointestinal tumors (Chakravarthy et al., 2018; Yang et al., 2020; Cui et al., 2021). At present, researchers have discovered many biomarkers for the diagnosis and treatment of gastrointestinal cancer, including human epidermal growth factor receptor2 (HER2) (Li Z. et al., 2020), mismatch repair deficiency/microsatellite instability (dMMR/MSI-H) (Dhakras et al., 2020), and programmed death-ligand 1 (PD- L1) (Dai et al., 2021). In addition, there are many new biomarkers under investigation, including neurotrophic-tropomyosin receptor kinase (NTRK) (Westphalen et al., 2019), claudin-18 (CLDN18) (Zhang et al., 2020), Rho GTPase-activating protein 26 (ARHGAP26) (Dhakras et al., 2020), fibroblast growth factor receptor (FGFR) (Babina and Turner, 2017), lymphocyte-activation gene 3 (LAG3) (Saleh et al., 2019), and T-cell immunoglobulin and mucin-domain containing-3 (TIM3) (Wang et al., 2017). However, only few clinical trials on UGIC patients have shown positive curative effects; the underlying mechanisms remain elusive so far. Nearly 50% of patients in good conditions will still suffer from local recurrence or systematic metastasis after aggressive treatment (Dhakras et al., 2020; Sung et al., 2021). It seems that most of the works aim at general tumor cells rather than cancer stem cells (CSCs) in UGIC. It is because CSCs are difficult to isolate due to the limitation of early experimental conditions and heterogeneity of CSCs (Clarke et al., 2006; Sreepadmanabh and Toley, 2018). Considering that the digestive tract organs share a common external environment and perform similar functions in a system, diet-induced mucosal lesions may have similar effects on cancer of the mouth, esophagus, and stomach (Haas et al., 2012). Therefore, it is necessary to take oral cancer, esophageal cancer, and gastric cancer as a whole, that is, UGIC, for integration research.

Some laboratories have conducted pan-cancer research on UGICs. Tran et al.'s pan-cancer study on somatic mutations found that leukocyte antigen-restricted T-cell receptors targeted the KRAS (G12D) hotspot driver mutation found in many human gastrointestinal cancers (Tran et al., 2015). Another study observed that IL-6 is the main communication medium for tumor cells and cancer-related fibroblasts in a murine model (Johnson et al., 2018). IL-6 deletion inhibits the occurrence of gastrointestinal tumors through STAT3 and MEK1/2 signals (Karakasheva et al., 2018). Dana–Farber Cancer Institute discovered the new immune checkpoint biomarker TET1 and PD-1 ligands (CD274 and PDCD1LG2) (Thienpont et al., 2016; Bu et al., 2021; Rahman et al., 2021). But fewer studies focus on CSCs. The problems of poor prognosis and a high recurrence rate still require more intensive studies in UGIC.

In this work, to verify the pathogenesis and therapeutic targets of UGIC, we performed the pan-cancer analysis on UGIC. Our results identified the unique CSCs in UGIG, which are named upper gastrointestinal common cancer stem cells (UGCSCs). The core regulation network of UGCSCs suggested that inflammation-related genes, namely, *CXCL8*, *PIGR*, and *CXCL3*, and Wnt pathway-related genes, namely, *GAST*, *REG1A*, *TFF3*, and *ZG16B*, are activated. Further analysis indicated that mucosal damage and inflammation caused by poor dietary habits trigger the Wnt pathway and eventually induce UGIC. In addition, GAST and TFF3 activate phosphatidylinositol 3-kinase (PI3k)/Ras to enhance the metastasis and invasion of UGIC. Taken together, these results pave the way for the better diagnosis and treatment of UGIC.

## Methods

### Data Collection and Processing

The data were collected from the published literature (Table 1). For different sequencing methods of single-cell data, specific analysis procedures were applied. For Drop-seq single-cell data, Cell Ranger software (Freytag et al., 2018) (3.0.1) was adopted to calculate the cell expression counts. For Smart-seq2 single-cell sequencing data, we operated cell expression matrixes provided in the original article. The expression matrix file was then imported into R 3.6.2 for subsequent analysis.

**TABLE 1:** Single-cell RNA-seq data of UGIC.

Species	Tumor type	Tissue	Sequence type	Cell number	Sample	PubMed ID
Human	EC	Esophagus	Smart-seq2	366	5	30223068 Wu et al. (2018)
Human	HNSCC	Oral cavity	Smart-seq2	4762	15	29198524 Puram et al. (2017)
Human	Early GC	Stomach	10x Genomics	4110	1	32209487 Zhang et al. (2019a)
Human	GC	Stomach	10x Genomics	29817	9	32532891 Zhang et al. (2021a)

### Data Normalization and Batch Effect Correction

First, we used Seurat (Stuart et al., 2019) (3.1.4) to filter the quality of cells and delete all cells with more than 6000 expressed genes or less than 201 genes. A total of 39,057 UGIC cells and 215,291 other cancer cells were obtained. Next, standardized integration processing was performed on the cell level, sample level, and study level.

#### Cell Level Standardization

The logarithmic percentage of gene expression in cells was adopted as the standardized integration of data between different cells in the sample (Butler et al., 2018). The value of the expression of gene x in a cell was divided by the value of the expression of all genes in this cell and multiplied by the scale amplification factor, which is set to 10000 in this experiment. Then, the logarithm of this value is the normalized value of the expression of gene x in the cell. This process can reduce the deviation of gene expression values caused by different sequencing depths and sequencing methods. The formula (1) is described as follows:

$$x_{i}^{\prime }=\log_{10}\left(\frac{x_{i}}{\sum_{i\in U}x_{i}}*10000\right),$$ (1)

where $x_{i}$ represents the expression value of gene i. $x_{i}^{\prime }$ represents the expression value of gene i after normalization. U represents the gene set in a certain cell.

#### Sample Level Standardization

We diminished gene features to avert the dimension disaster problem in the single-cell expression matrix. First, the logarithm of gene expression means and variances was calculated. Next, we fitted a line regression model to represent the relationship between the two values using the local polynomial regression. Next, we normalized the gene expression value through the mean value and expected variance of the model. Finally, the top variable 2000 gene features were selected for the subsequent analysis based on the normalized expression value.

#### Study Level Standardization

We conducted an integrated analysis of multiple samples, by looking for similar sites between cells. First, the dimensionality was reduced by using canonical correlation analysis (CCA) (Andrew et al., 2013). Next, similarity anchor points were constructed, according to the similarity of sample expression matrixes. Finally, the data were integrated between different studies, according to the identified anchor points.

### UGIC Cell Type Identification

After PCA dimensionality reduction was performed on 39,057 UGIC cells, nine cell sets were obtained by T-distributed stochastic neighbor embedding (t-SNE) clustering. In order to identify the cell types, we calculated highly expressed genes on each cluster through the FindMarkers (Butler et al., 2018) function in Seurat. Then, through the artificial gene annotation on the CellMarker (RRID:SCR_018503) database (Zhang X. et al., 2019), the marker genes and the corresponding cell type were finally annotated. We show the statistical graph of cell types identified by EPCAM in the published articles as an example in the CellMarker (RRID:SCR_018503) database (Supplementary Figure S5). Then, we analyzed the subtypes of cancer stem cells, obtained a total of six subclasses, and calculated the differentially expressed genes (DEGs) of each subclass.

### Other Cancer Cell Type Identification

A total of 71 single-cell sequencing data (Supplementary Table S2) from six other cancers were collected. We used the same method to process other tumor single-cell data to ensure the consistency of the analysis process. First, the quality control of single-cell data obtained a total of 215,291 cells. After standardization at the cell level, sample level, and study level, we used PCA and t-SNE visualization to reduce the dimension of those single-cell data and obtained 29 cell collections. We calculated the highly expressed genes of 29 cell collections and used the CellMarker database (Zhang X. et al., 2019) to annotate the cell types. Then, we marked cancer stem cells, which are subtypes 4 and 7. The relevant marker annotations are shown in Supplementary Figure S3.

### UGIC Transcriptome Sequencing Analysis

We gathered bulk RNA-seq data of UGICs in the TCGA database (Aldape et al., 2015). We obtained the expression matrix data using the cBioPortal (Cerami et al., 2012; Gao et al., 2013), including 522 HNSCC samples, 185 EC samples, and 415 GC samples. Three types of UGICs were congregated with the data label “hnsc_tcga,” “esca_tcga,” and “stad_tcga”. DESeq2 (RRID:SCR_000154) (Love et al., 2014) (1.26.0) software was used to measure the DEGs in the cancer sample and the corresponding normal sample.

### Gene Function Annotation

We annotated the function and pathway information of the significantly different genes in the Gene Ontology (GO) (Ashburner et al., 2000; Ashburner, 2021) database and Kyoto Encyclopedia of Genes and Genomes (KEGG) (RRID:SCR_012773) (Kanehisa et al., 2021) database using the clusterProfiler (RRID:SCR_016884) (Yu et al., 2012) (3.14.3) package in R (3.6.2) software. The top 15 terms are presented in Figure 4.

### Gene Enrichment Analysis

We adjusted the gene set enrichment score between the specific differential genes and the cancer-related gene sets through the Gene Set Enrichment Analysis (GSEA) (Subramanian et al., 2005) software (4.1.0). “C6: oncogenic signatures” was selected as the existing cancer-related gene set in GSEA software. We filtered several parameters to draw gene enrichment results. The normalized enrichment score (NES) was larger than 1. The normalized significance level (NSL) *p*-value was lower than 0.05.

### Protein Interaction Network Analysis

We collected all human entries in the String database (RRID:SCR_005223) and deleted low-quality and text mining entries (Szklarczyk et al., 2021). After removing the duplicated edges and self-loops, we constructed a human protein–protein interaction network (PPIN) with 19,267 proteins and 1,689,887 edges by Cytoscape software (RRID:SCR_003032) (Shannon et al., 2003) (3.7.1). Then, 174 genes specifically expressed in UGCSC were mapped to the PPIN. After removing outlier proteins, a regulatory sub-network composed of 144 protein nodes and 545 edges was constructed. Next, we appraised the topological attributes of the network and selected the degree and clustering coefficient (CC) to measure the function of the sub-network (Sporns, 2013). The degree represents the number of connections through a particular node, which measures the importance of the node in the network. CC represents the closeness of connections between a node and the surrounding nodes, which demonstrates the network closeness and function similarity. The formula (2) is described as follows:

$$C_{i}=\frac{2e_{i}}{d_{i}\left(d_{i}-1\right)},$$ (2)

where $C_{i}$ represents the CC of gene i. $d_{i}$ represents the count of adjacent nodes of gene i. $e_{i}$ represents the number of interconnected nodes among all adjacent nodes of gene i.

### Construction of the Hub Gene Function Network

We manually reviewed the tumor-related literature studies published since 2000 to screen functions and pathways of DEGs. Then, we formulated UGCSC function networks by integrating different genes and the known inflammation and Wnt pathways (Figure 6).

## Result

### Landscape of UGIC Single-Cell Data

**FIGURE 1:**
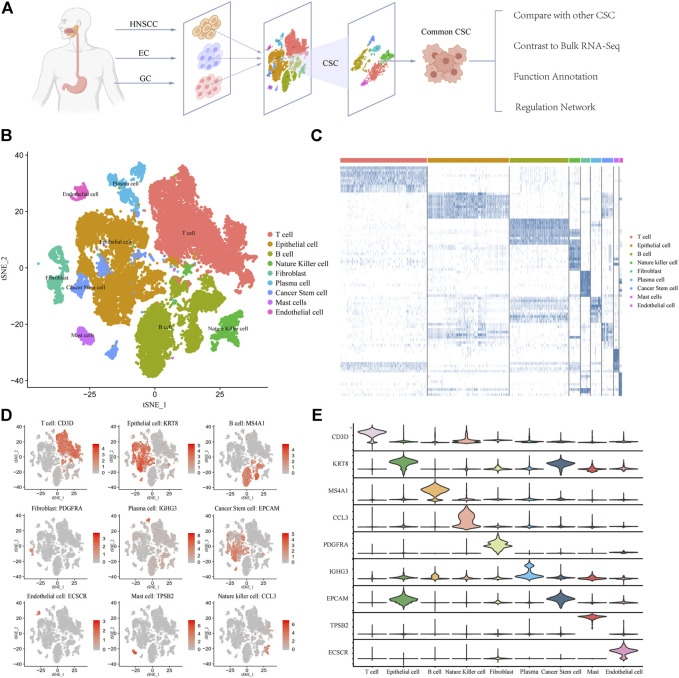
Expression profiling of 39,057 single cells in UGIC. **(A)** Workflow of sample processing, cell type annotation, and functional analysis for 30 samples in UGIC. **(B)** t-SNE of 39,057 cells profiled here, with each cell color-coded for the associated cell type. **(C)** Heatmap of the expression pattern in each cell type. **(D)** Expression of marker genes for the cell types defined above each panel. **(E)** Expression trend of marker genes for each cell type in the violin chart.

We collected 39,057 tumor single-cell sequencing data from 30 patients including 4762 HNSCC cells, 366 EC cells, and 33,927 GC cells (Figure 1A). It is noteworthy that EC samples applied Smart-seq2 single-cell sequencing technology (Picelli et al., 2014) which is manually sequencing each cell. So the EC group has few cells but higher confidence. After quality filtering (see Methods) and removing the batch effect, more than 70 million transcripts were obtained from 39,057 cells. Subsequently, we classified cells into different clusters by using T-distributed stochastic neighbor embedding (t-SNE) methods in Seurat software (Supplementary Figure S1). Through marker genes, these identified cell clusters could be assigned to known cell lineages: T cells, B cells, epithelial cells, natural killer cells, fibroblasts, plasma cells, cancer stem cells, mast cells, and endothelial cells (Figure 1B). To corroborate these profiles, we showed the high expression gene distribution heatmap of each cell type and the expression abundance of marker genes of each type (Figure 1C; Supplementary Table S1). Each cell type has specific marker genes: *CD3D*, *KRT8*, *MS4A1*, *PDGFRA*, *IGHG3*, *EPCAM*, *ECSCR*, *TPSB2*, and *CCL3* (Figure 1D). The violin plot of marker genes shows that the expression of most marker genes is specific, which indicates that the classification of cell types is accurate and is very helpful for subsequent analysis (Figure 1E). Taken together, these results indicate that the cell classification was accurate, and most of the cells were classified into the correct cell type. The distribution of samples and cancer types is shown in Supplementary Figures S1, S2. We also counted the number and frequency of all cell types in HNSCC, EC, and GC and provided the results in Supplementary Figure S6.

### UGIC-Specific Cancer Stem Cell Identification

**FIGURE 2:**
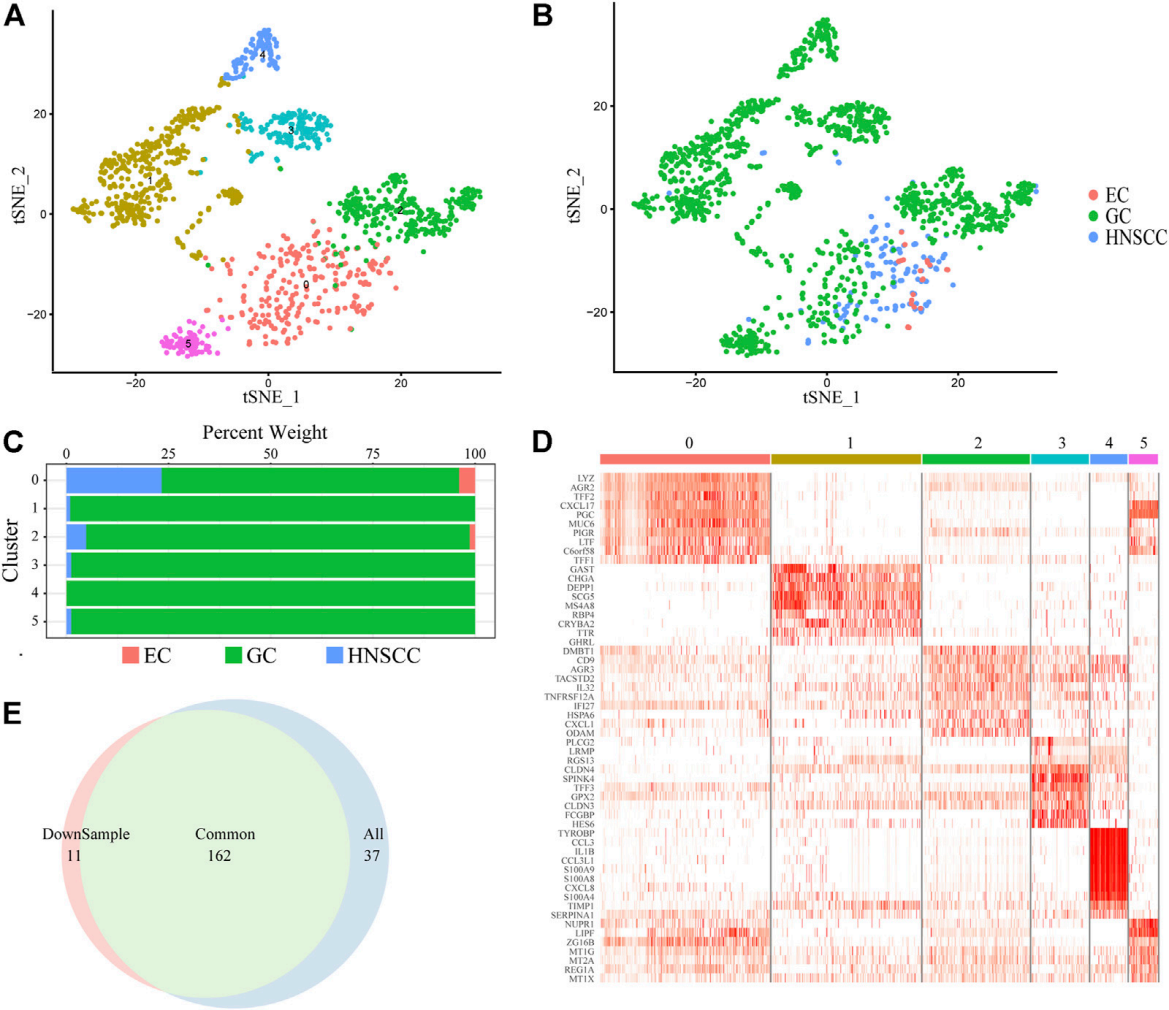
Cancer stem cell clusters. **(A)** t-SNE plot of 1,586 CSC cells color-coded by sub-clusters. **(B)** t-SNE plot color-coded for cancer type of origin. **(C)** Histogram of cell numbers in each CSC cancer type. **(D)** Heatmap of the expression pattern of six sub-clusters in CSC. **(E)** Venn diagram of DEGs in downsample CSCs and All CSCs.

We focused on cancer stem cell types in order to reveal the pathogenesis and distant metastasis mechanism of UGIC. We collected a total of 1,586 CSCs (Figures 2A,B) including 136 HNSCC cells, 23 EC cells, and 1427 GC cells. Due to the heterogeneity of CSCs, there are differences in the same type of cancer while similarities exist in different types of cancers, coincident with the characteristics of the remote metastasis and recurrence of the cancers. Therefore, we performed a cluster analysis of CSCs, and a total of six sub-clusters were found. After annotating and analyzing all sub-clusters, sub-cluster 0 is ubiquitous in UGICs, including 19 EC stem cells, 356 GC stem cells, and 114 HNSCC stem cells, which proves that sub-cluster 0 preliminarily meets the characteristics of common CSCs (Figures 2C,D). Therefore, we concentrated on sub-cluster 0 in the follow-up analysis.

To verify whether sub-cluster 0 reflects the characteristics of UGIC rather than only GC, we performed a down-sampling process in sub-cluster 0 since there are more than 70% GC stem cells in sub-cluster 0. We randomly selected the same number of GC cells as the HNSCC cells and named new sub-cluster 0. Subsequently, we compared the differential genes between sub-cluster 0 and the new sub-cluster 0 in CSCs. The merge ratio is 77.14% (Figure 2E), which means these two sub-clusters share the same differential gene set. These results indicate that the differentially expressed genes (DEGs) of sub-cluster 0 represent the features of UGICs.

**FIGURE 3:**
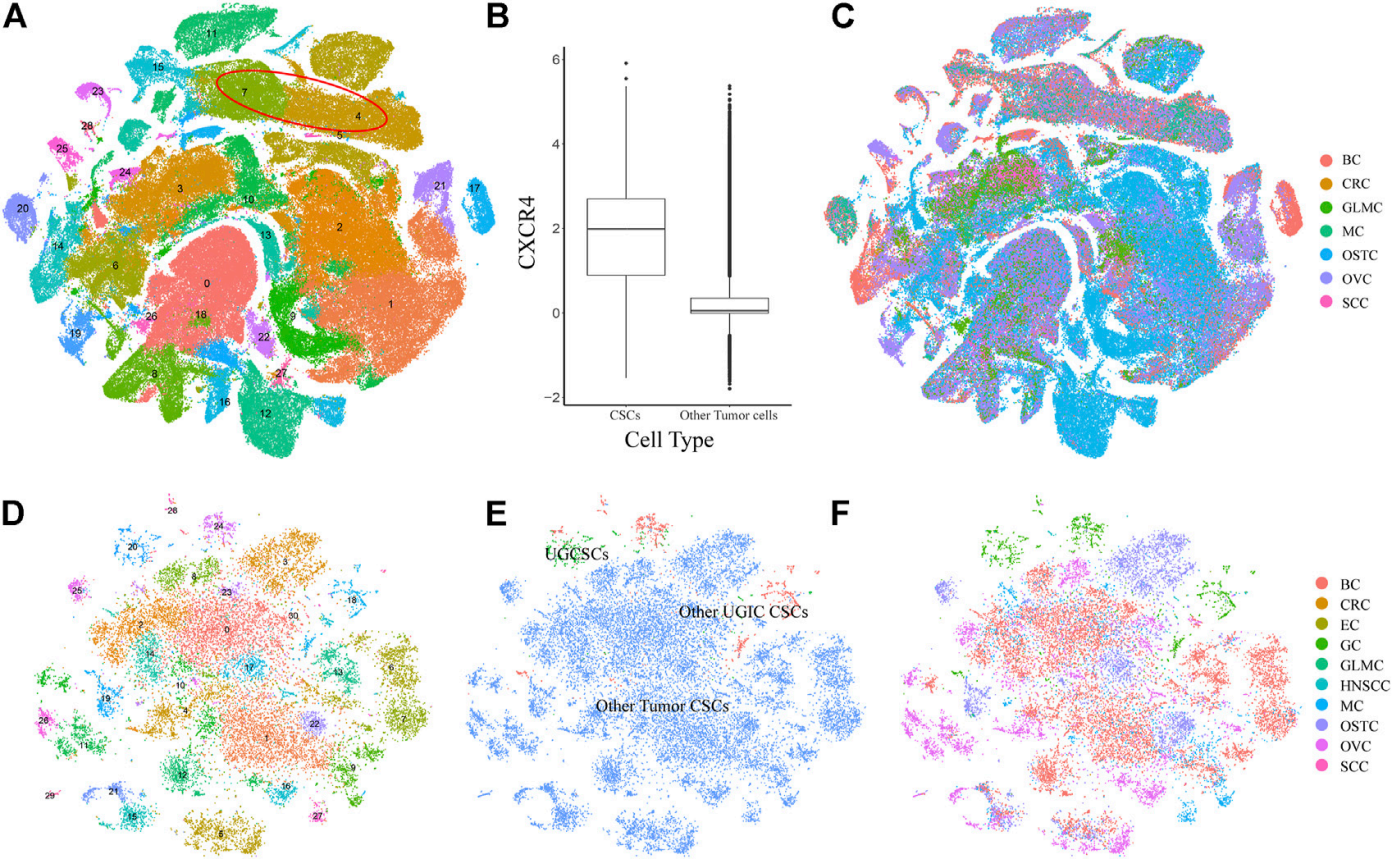
Expression profiling of 215,291 single cells in six cancer types. **(A)** t-SNE plot of 215,291 single cells in six cancer types, color-coded by 29 clusters. Clusters 4 and 7 are cancer stem cells, which are marked by a red circle. **(B)** Box plot shows the expression of the CSC marker gene *CXCR4*. The *x*-axis represents the cell type. The *y*-axis represents the log value of the normalized CXCR4 expression. **(C)** t-SNE plot of other cancer cells, color-coded by the cancer type. **(D)** t-SNE plot of all CSCs, color-coded by clusters. **(E)** t-SNE plot of all CSCs, color-coded by the CSC type. **(F)** t-SNE plot of all CSCs, color-coded by cancer types.

To validate the specificity of UGCSCs, we compared UGCSCs with other tumor cells. We collected 71 samples (215,291 cells) from six types of cancers including glioma (GLM), melanoma (MELA), osteosarcoma (OSTC), breast cancer (BC), ovarian cancer (OVC), and stellate cell cancer (SCC) (Supplementary Table S2). After normalizing the cells and removing the batch effect (see Method), all the cells were gathered into 29 sub-clusters (Figures 3A,C). After annotating all cancer cells in the CellMarker database, we noticed that there are plenty of cell types due to the complexity of tissue types involved. Therefore, we only annotated CSCs by using marker genes. The tumor stem cells were obviously aggregated with CXCR4 markers (Figure 3B; Supplementary Figure S3), which are sub-clusters 4 and 7 and contain 21323 cells, as circled in Figure 3A. We compared CSCs of other cancers with CSCs of UGICs. We re-clustered and obtained 31 sub-clusters in all CSCs, which reveals the differences between CSCs of different tumor types (Figure 3D). But at the same time, the cluster distribution of CSCs from different tissues is uniform, which indicates that there are similarities between different tissues in CSCs (Figures 3E,F). This phenomenon is also coincident with the heterogeneity of tumors. The cluster annotation of cancer types shows that the UGCSC is self-clustering and far away from other tumor CSCs (Figure 3E). Therefore, the UGCSC is the specific cancer stem cell in UGIC while UGCSC does not exist in other cancers.

### UGCSC Function Analysis

**FIGURE 4:**
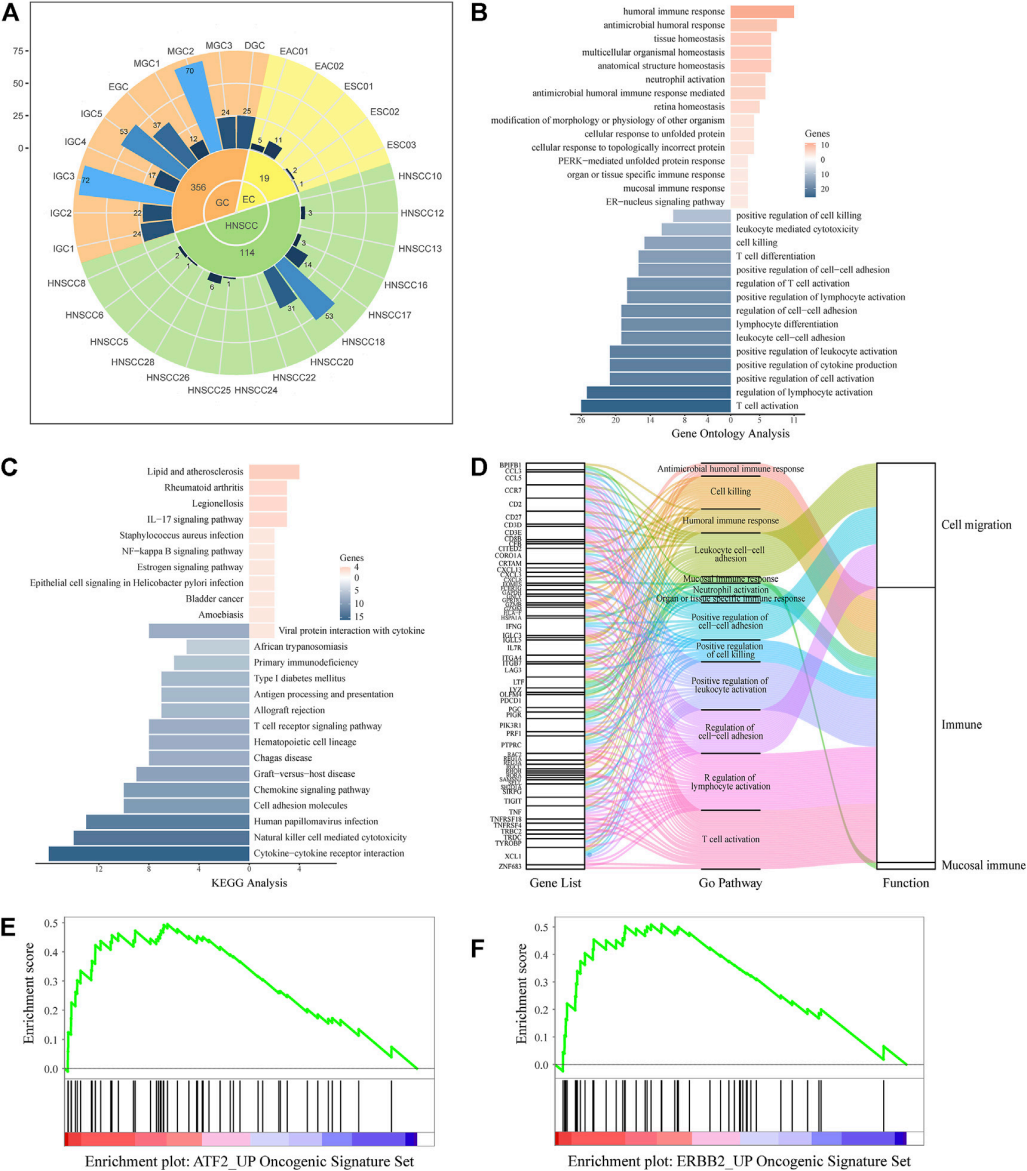
UGCSC function annotation. **(A)** Sample source of UGCSCs. Green, yellow, and orange represent HNSCC, EC, and GC, respectively. The inner circle is for three types of cancer, and the middle circle is the number of cells in each cancer. The outer circle represents the number of cells in each sample. **(B)** Gene ontology function annotation of UGCSC DEGs. **(C)** Pathway analysis of UGCSC differentially expressed genes. **(D)** Gene function integration in UGCSCs. **(E)** UGCSC differentially expressed gene set enrichment analysis in the ATF2_UP tumor set. **(F)** UGCSC differentially expressed gene set enrichment analysis in the ERBB2_UP tumor set.

We comprehensively analyzed the distribution and function of UGCSCs. The cell sources of UGCSC cancers were analyzed and counted (Figure 4A). As shown in Figure 4, UGCSCs are averagely expressed in UGIC patients, including 10 GC patients, four EC patients, and nine HNSCC patients. In summary, the UGCSCs are distributed uniformly, which proves that the UGCSC is common in upper gastrointestinal patients.

We analyzed the expression network of UGCSCs. First, we compared the expression profiles of UGCSCs and all other tumor stem cells and obtained 174 genes with significant differences, including 33 upregulated genes and 141 downregulated genes (Supplementary Data S1). We uncovered that the gene information function reflects the characteristics of UGCSCs as a digestive system and as cancer stem cells by analyzing the function annotation (Ashburner et al., 2000) of DEGs (Figures 4B,D). The upregulated genes are related to antibacterial response, such as “antibacterial humoral response,” “antibacterial humoral immune response-mediated,” and “mucosal immune response,” which are consistent with the function of the digestive tract in the human body. In addition, the functions of downregulated genes mainly focus on reducing the activity of T cells and lymphocytes and downregulating cell killing which could reduce the body’s immune response and enhance the survival rate of tumor cells, and these are also the characteristics of cancer stem cells. The downregulated genes also play a role in the regulation of cell–cell adhesion to facilitate the distant metastasis of tumors, which is in line with the feature of metastasis. Through the analysis of the KEGG pathway (Kanehisa et al., 2021), we observed that the significantly differentially expressed genes are enriched in inflammation-related mucosal infections such as “*Staphylococcus aureus* infection,” “epithelial cell signaling in *Helicobacter pylori* infection,” “IL-17 signaling pathway,” and “chemokine signal pathway” (Figure 4C). These results uncovered a potential carcinogenic factor of UGIC, that is, mucosal damage induced activation and mutation in inflammatory pathways. Through gene set enrichment analysis (GSEA), we found that the significantly differentially expressed genes were significantly enriched in ATF2- and ERBB2-related cancer genes (Figures 4E,F). To further confirm the reliability of these genes, we calculated the DEGs between UGCSCs and 30,267 cells of normal tissues in the upper alimentary tract (Cillo et al., 2020; Zhang X. et al., 2021) (Supplementary Figure S7A). The DEGs of UGCSCs and tumor cells are few and share fewer genes with the DEGs of UGCSCs and normal cells (Supplementary Figure S7B). Also, the functional analysis of DEGs of UGCSCs and normal cells indicates that the functional pathways are related to cell development, which is a common feature of tumors (Supplementary Figure S7C). In summary, these results indicate that the DEGs of UGCSCs and tumor cells are oncogenes related to the function of the digestive tract.

### UGIC Carcinogenic Mechanism Detection

**FIGURE 5:**
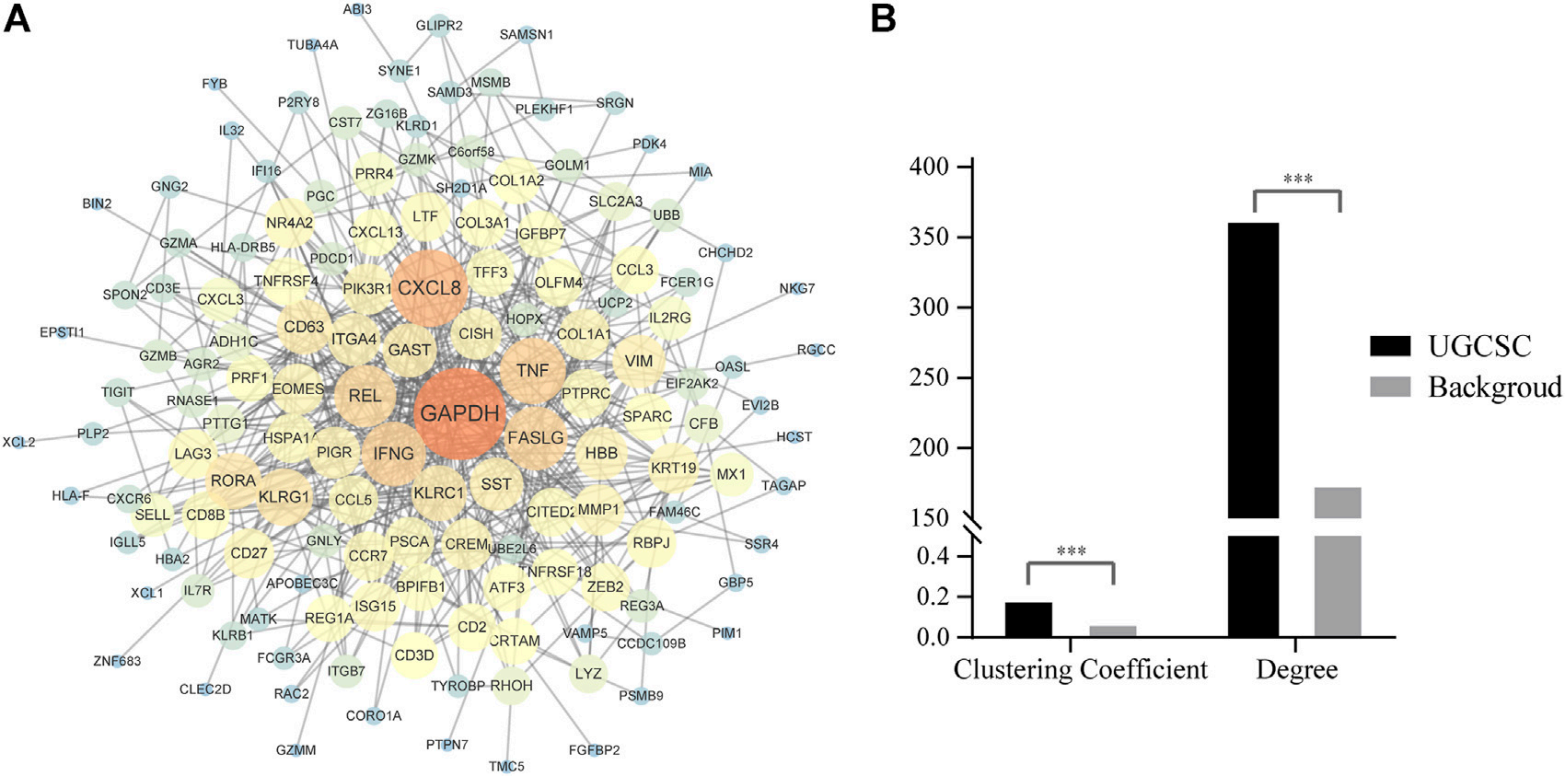
Network analysis of UGCSCs. **(A)** Protein regulation networks of DEGs in UGCSCs. **(B)** Topological attribute comparison of UGCSC sub-networks and whole PPIN.

In order to study the pathogenic mechanisms that may exist in UGCSCs, we mapped 174 proteins into the human protein–protein interaction network (PPIN). We constructed PPIN and deleted low-quality text mining terms in the String database (Szklarczyk et al., 2021). After mapping 174 DEGs in UGCSCs into PPIN, an interaction network consisting of 144 proteins and 545 edges was obtained (Figure 5A). Through the analysis of the topological properties of the network, we found that the degree of DEGs in PPIN is 362.174, which is significantly higher than 175.418 in the human total network (Figure 5B). This result indicates that the shortest path through different genes is significantly higher than the average value (Figure 5B), which implied that these genes are hub genes in the UGCSC network. Furthermore, another topological property, the clustering coefficient (CC), is significantly higher than the background network, which points out that the 144 genes are closely linked compared with the random gene set in the network. The close interaction means a similar or synergistic function in cells. Through the comprehensive analysis of degree and CC, we inferred that the 144 genes are tightly connected hub genes in PPIN, which means that they play an important function in UGCSCs as a co-operative hub gene set.

**FIGURE 6:**
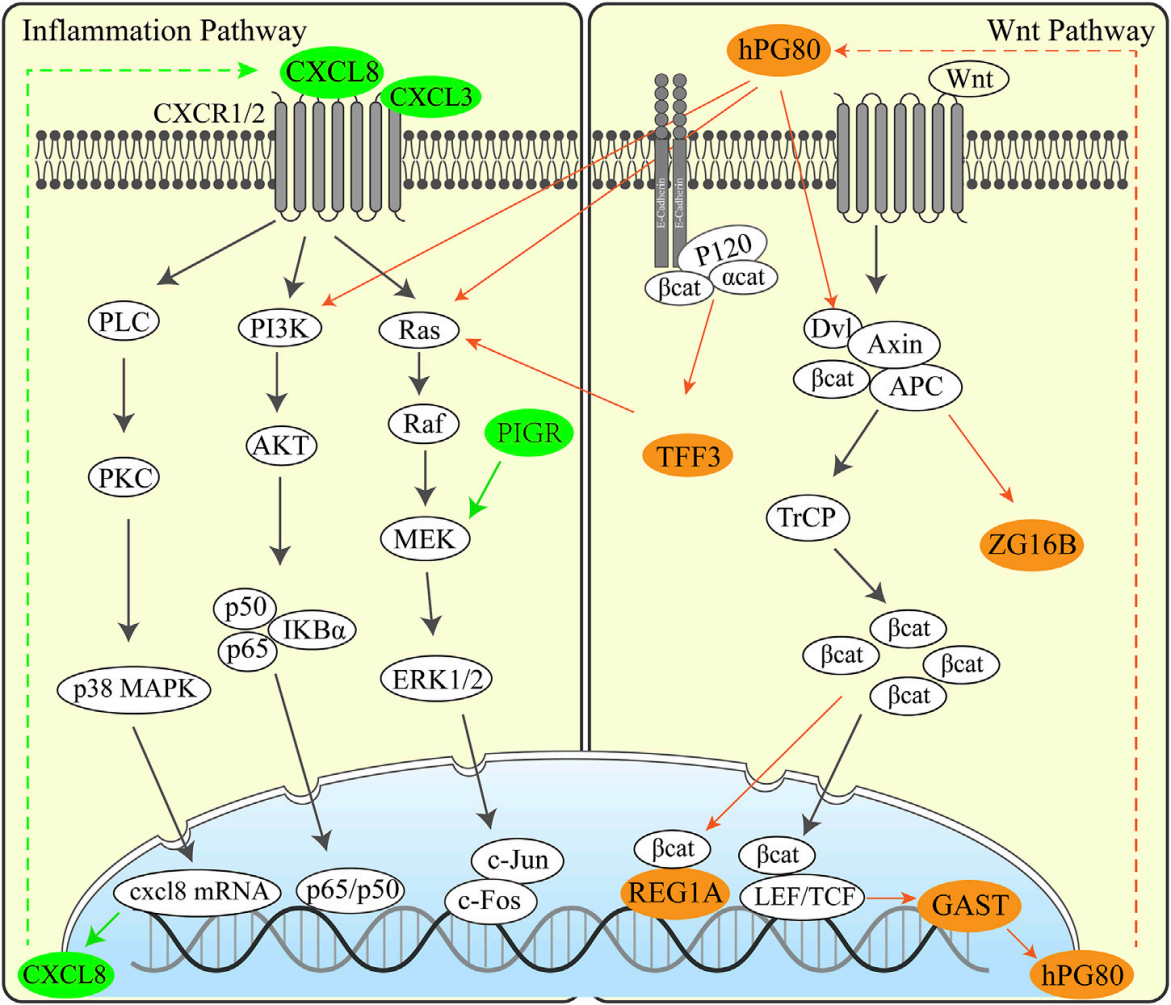
Regulation pathway in UGCSCs. Inflammatory pathway (left) and Wnt pathway (right) activated in UGCSC. The green genes represent inflammatory factors that are highly expressed in UGCSCs. The brown–yellow genes represent Wnt-related factors that are highly expressed in UGCSCs.

We have performed functional annotations on the possible functions of these genes and inferred regulatory pathways with the aim to explore the possible pathogenic mechanisms and potential therapeutic targets in UGIC. We analyzed the regulation pathway of those genes through published articles and proved that the upregulated genes are basically related to cancer (Supplementary Table S3). Here are some exciting discoveries. Some genes are related to inflammatory pathways, such as CXCL8 (Ha et al., 2017), BPIFB1 (Li J. et al., 2020), PIGR (Kakiuchi et al., 2020), CXCL3, and RNASE1 (Wang et al., 2006), and some genes are related to specific functions of the digestive tract, such as GAST (Giraud et al., 2016), REG1A (Sha et al., 2019), and TFF3 (Braga Emidio et al., 2020). These results illustrated that there may have similar pathogenic mechanisms and common regulatory pathways in some UGICs. We speculated that mucosal damage is induced by long-term unhealthy eating habits, which include smoking, drinking, and hot food breed inflammation. Persistent inflammation leads to carcinogenic mutations and early gastrointestinal tumors. These conjectures have been confirmed in the specific regulatory network of UGCSCs. Based on the detected differentially expressed genes and the mining of relevant research literature studies, we speculated the pathogenesis of the disease, as shown in the Figure 6. Inflammation-associated interleukin (CXCL8 and CXCL3) and inflammation defense-related BPIFB1, PIGR, and RNASE1 are activated in UGCSCs. Combined with the epidemiological investigation of gastrointestinal cancer, there is a hypothesis that chronic inflammation is incited by mucosal damage due to long-term bad eating habits. We present that the cancerous chronic inflammation is activated by GAST, REG1A, TFF3, and ZG16B in the Wnt signaling pathway. Upregulated hPG80 and TFF3 induce PI3K/Ras and lead to tumor cell growth and invasion, which may be one reason for the poor prognosis of UGIC. Hence, this resource provides a novel view for the occurrence and development of UGIC and the advancement of gastrointestinal cancer diagnosis and therapy.

## Conclusion

In this article, we collectively analyzed single-cell sequencing data of HNSCC, EC, and GC and identified a specific cancer stem cell type in UGIC: UGCSCs. Then, we presented the unique expression pattern and hub gene set in UGCSCs by comparing it with other tumors’ single-cell RNA-seq data. We declared the common carcinogens of UGICs that the mucosa damage of the digestive tract induces chronic inflammation due to unhealthy eating habits. The hub gene set provides promising entry points for the design of novel therapies including CXCL8, CXCL3, GAST, TFF3, PIGR, and RNASE1.

## Discussion

Here, we provided a comprehensive catalog of human UGICs at single-cell resolution. In the integrative analysis of UGICs, we confirmed that there are specific cancer stem cells in UGIC, which are named UGCSCs. This discovery provides a new perspective for scientific analysis of the poor prognosis and easy recurrence of UGIC. By comparing the tumor stem cells of six cancers, we extracted the core gene set that plays an important role in UGCSCs and explored the possible pathogenic pathway of UGIC and core genes including *GAST*, *CXCL8*, *CXCL3*, *PIGR*, *REG1A*, and *TFF3*. With further in-depth research, these genes can also be used as diagnostic markers or possible therapeutic targets for gastrointestinal cancers.

However, all cell types and subtypes cannot possibly be described here; some key results emerge. On one hand, the distribution of all cell types in UGIC is shown in the cell clustering figure (Figure 1B). On the other hand, through the comparative analysis with bulk RNA-seq sequencing data, the DEGs between single-cell data and bulk RNA-seq data varied significantly. Therefore, we performed further research only on cancer stem cells. Intriguing questions remain as to whether there are specific immune cells in UGIC and whether the immune cell counts would have an impact on the prognosis of UGIC.

The single-cell data of UGIC and the six cancer types are composed of cells from different patients. Some sub-clusters of cell types have different abundances due to sample differences, according to the results of cell clustering. We removed batch effects and deleted outlier cells from the clustering result. In this way, the impact of samples from different patient sources is reduced.

We performed the same analysis on bulk RNA sequencing data; however, due to the varieties of cell types and the low proportion of CSCs in cancer tissues, the pathways and therapeutic targets were not discovered. We collected 1,122 patients and 1,966 normal samples of bulk RNA-seq data in TCGA database. The differentially expressed genes of the three cancers were compared with those of UGCSCs. The result suggests that the merge ratio is only 0.79%. Moreover, the function of differentially expressed genes is mostly about cell cycle-related pathways in bulk RNA-seq data (Supplementary Figure S4). We inferred that plenty of cell types in UGIC generates noises in UGIC expression profile information and makes some core pathways and genes undetectable, while single-cell RNA-seq can filter noise signals by extracting specific cell types.

Last, we constructed a regulatory network of UGCSCs under the framework of the existing experimental knowledge atlas. More and other types of data such as downstream genes and mutation information of the core regulatory network need to be further studied. However, we proposed UGCSCs and their regulatory networks based on the analysis of single-cell data from more than 100 patients and more than 25,000 cells, which has strong robustness. These data build a framework for a deeper understanding of the molecular mechanisms of UGCSCs and the regulation network of hub genes and might be applied to screen for molecular target drugs to improve the efficacy and outcomes for UGIC patients.

## Data Availability

The UGIC single-cell sequencing data are available through SRR6133148 (Wu et al., 2018), GSE103322 (Puram et al., 2017), and GSE134520 (Zhang et al., 2019a) in the Gene Expression Omnibus (GEO) and HRA000051 (Zhang et al., 2021a) in Genome Sequence Archive (GSA). The normal single cell data of upper alimentary tract are available through GSE139324 (Cillo et al., 2020) and GSE160269 (Zhang et al., 2021b) in the GEO database and HRA000051 (Zhang et al., 2021a) in GSA database. The other cancer single-cell sequencing data are available through GEO database: GSE152048 (Zhou et al., 2020), GSE89567 (Venteicher et al., 2017), GSE72056 (Tirosh et al., 2016), GSE75688 (Chung et al., 2017), GSE84465 (Darmanis et al., 2017), and GSE102130 (Filbin et al., 2018) in GEO database and https://lambrechtslab.sites.vib.be/ (Qian et al., 2020) website. Gene interactions networks were identified using the STRING database (https://string-db.org) (Szklarczyk et al., 2021). GO term analysis was performed by using the GENEONTOLOGY database (http://geneontology.org/) (Ashburner et al., 2000). Tumor gene sets enrichment analysis was performed using the MSigDB database (www.gseamsigdb.org) (Subramanian et al., 2005). All other data are included in the article and its Supplementary Information files or available from the corresponding authors upon reasonable request.
