# Supplementary material for: Pan-Cancer Single-Cell Analysis Reveals the Core Factors and Pathway in Specific Cancer Stem Cells of Upper Gastrointestinal Cancer
Source: Front Bioeng Biotechnol. 2022 May 13;10:849798. doi: 10.3389/fbioe.2022.849798 (PMC9136039; doi:10.3389/fbioe.2022.849798)
Supplement: Supplementary file 2 [file DataSheet1.PDF]

Pan-cancer analysis reveals the core factors and pathway in cancer stem cells of upper gastrointestinal cancer

Leijie Li[1], Yujia Zhang[1], Yongyong Ren[1], Zhiwei Chen[1], Yuening Zhang[1], Xinbo Wang[1], Hongyu Zhao[2], Hui Lu[1].

1 SJTU-Yale Joint Center for Biostatistics and Data Science, School of Life Sciences and Biotechnology, Shanghai Jiao Tong University, Shanghai, China.
2 Department of Biostatistics, Yale University, New Haven, CT, USA
* To whom correspondence should be addressed. email: huilu@sjtu.edu.cn

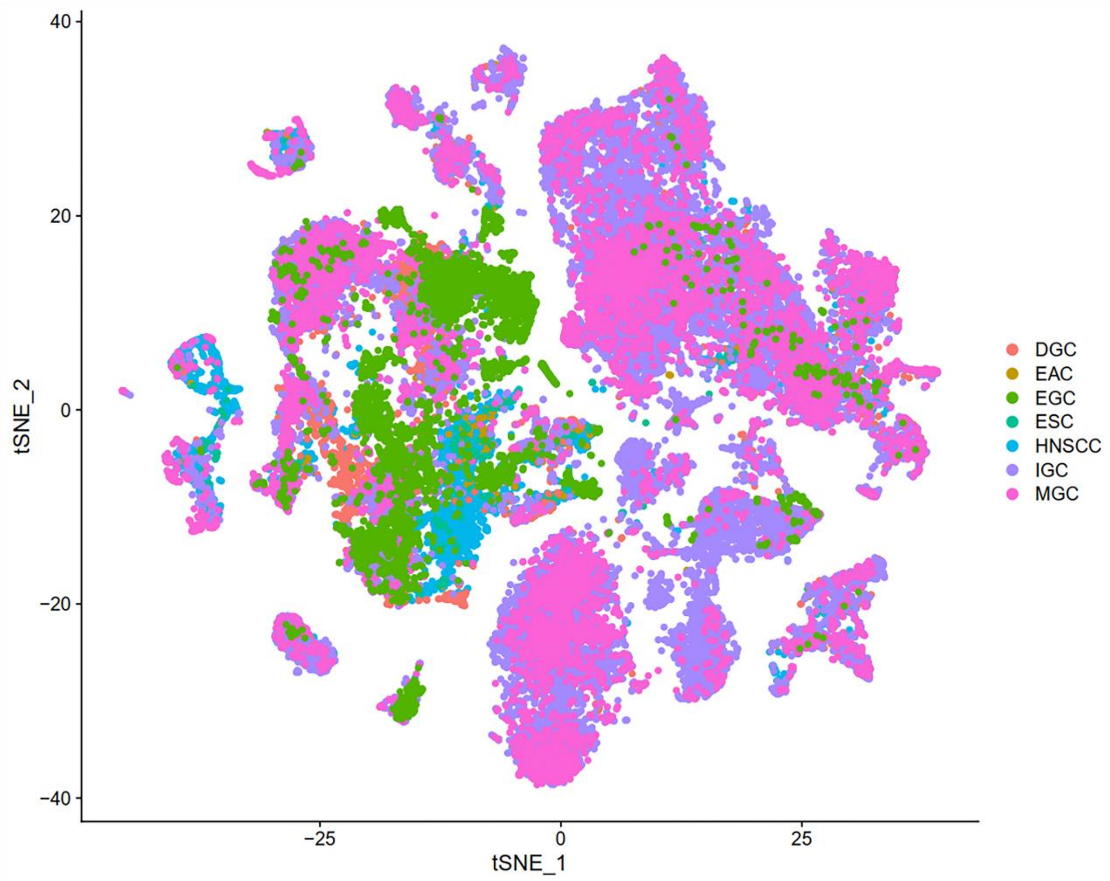

Supplementary Figure 1 Cancer type distribution of UGIC t-SNE plot

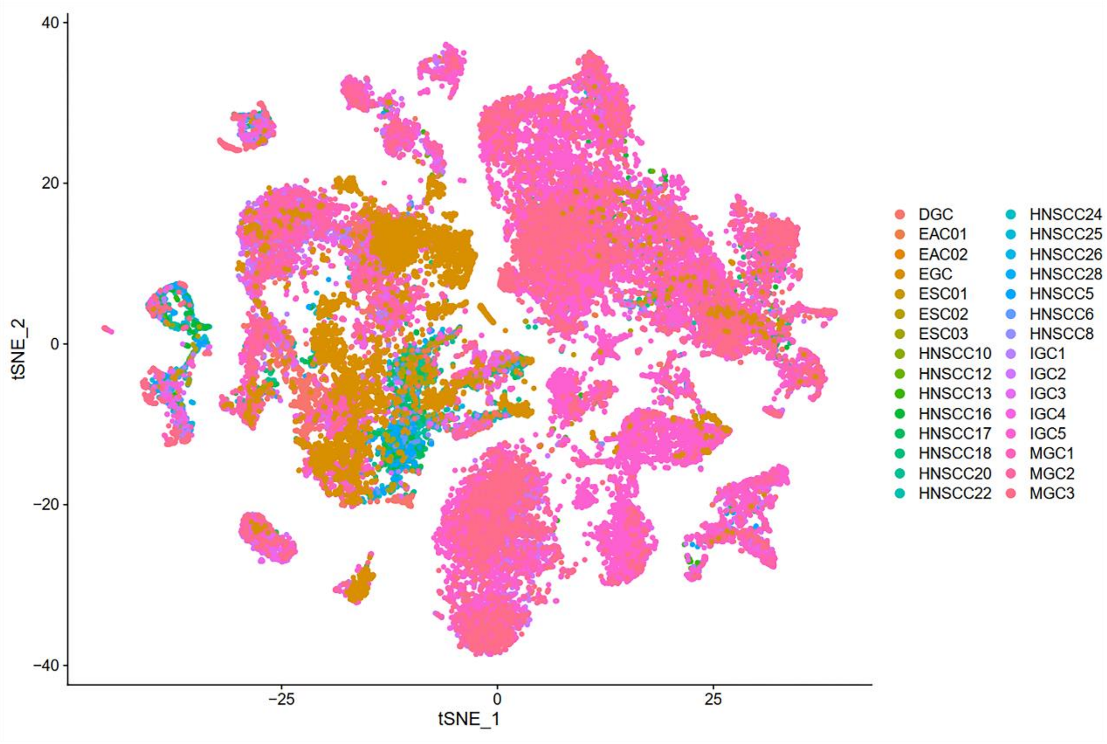

Supplementary Figure 2 Sample distribution of UGIC t-SNE plot

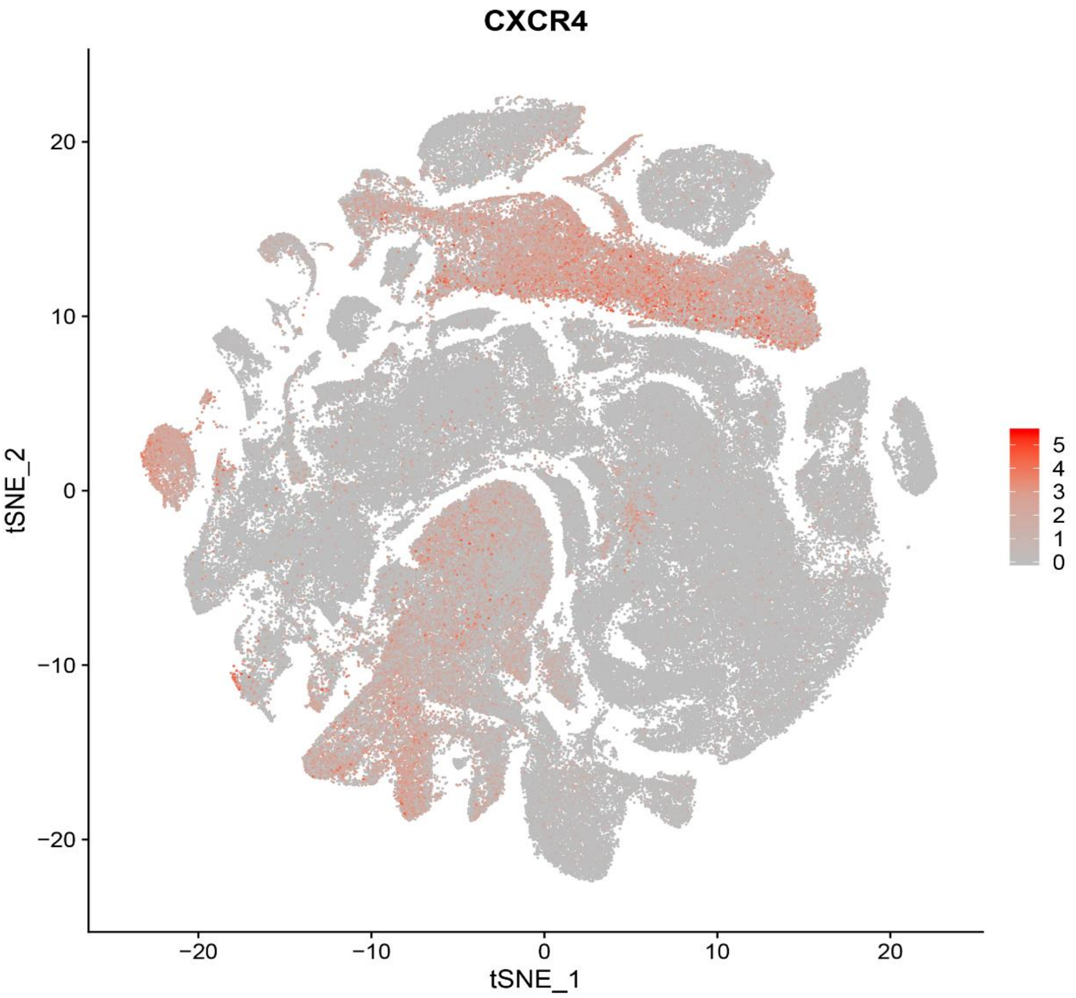

Supplementary Figure 3 Expression pattern of CXCR4 in other cancers.

A

Cancertype	Cancer Sample	Normal Sample
HNSCC	522	162
EC	185	1445
GC	415	359

B

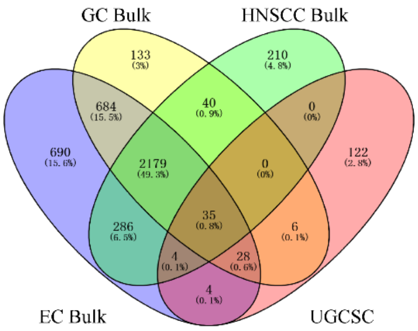

C

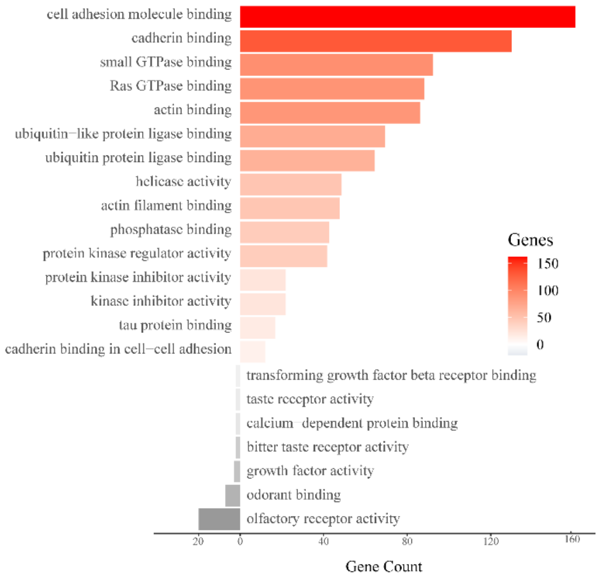

Supplementary Figure 4 Bulk RNA-seq analysis of UGIC

(A) The number of samples in HNSCC, EC, and GC. **(B)** Venn diagram of differentially expressed genes in bulk RNA-seq and UGCSC. **(C)** GO function annotation of common significant differentially expressed genes in bulk RNA-seq of UGIC.

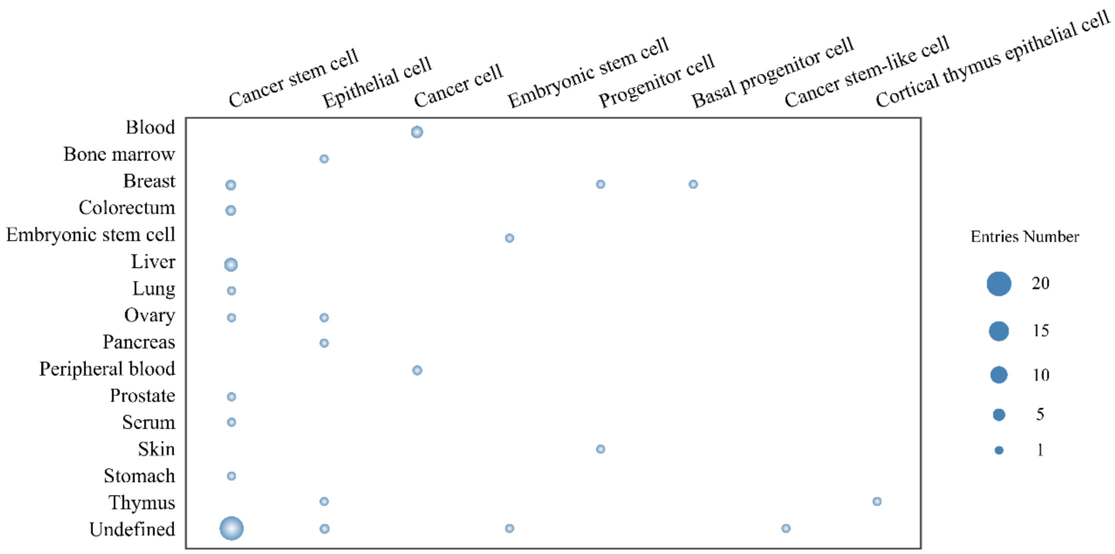

Supplementary Figure 5 Statistical graph of cell types identified by EPCAM in published papers.

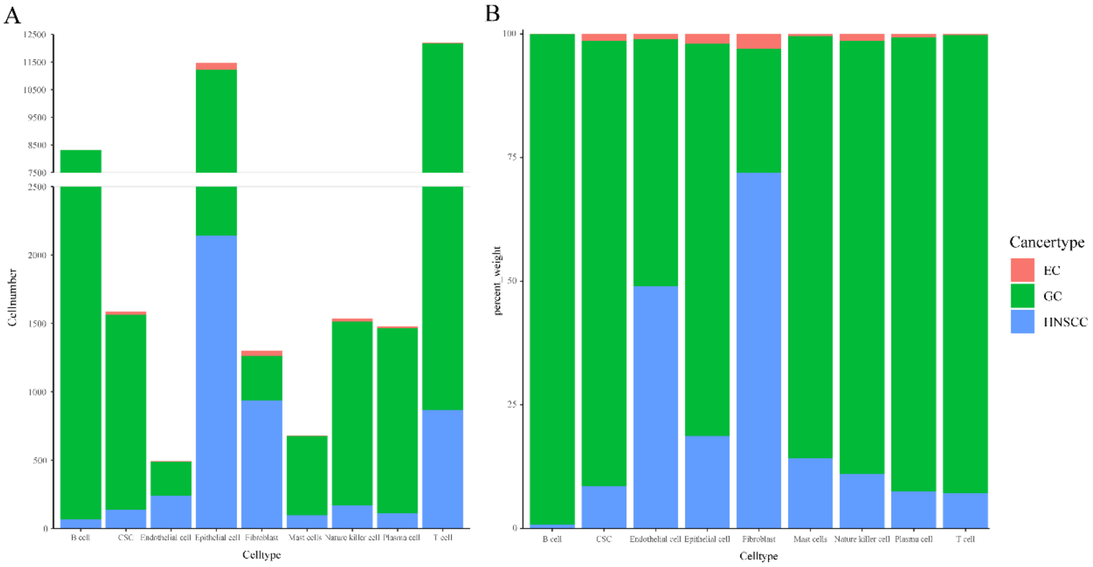

Supplementary Figure 6 Cell numbers and frequency of all cell types in HNSCC, EC, and GC. **(A)** The cell number of all cell types in HNSCC, EC, and GC. **(B)** The cell frequency of all cell types in HNSCC, EC, and GC.

A

Tissue	States	Cell	Sample	PMID
Amygdale	Normal	12861	5	31924475
Esophagus	Normal	11505	4	34489433
Stomach	Normal	5901	2	32532891

B

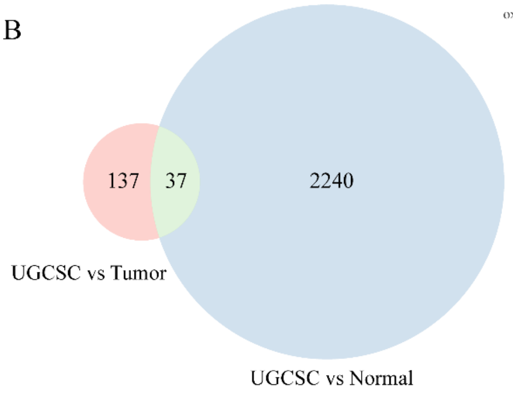

C

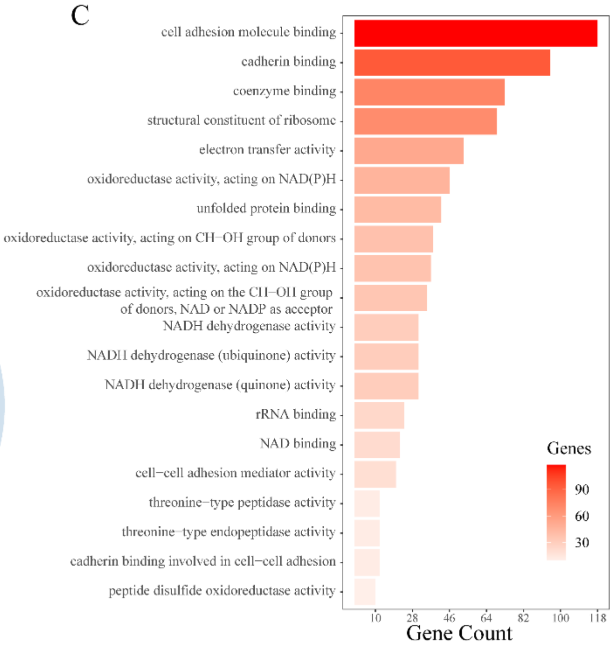

Supplementary Figure 7 DEGs of UGCSCs and normal single cells.

(A) Normal Sample and cell number in upper alimentary tract. **(B)** Venn of DEGs in UGCSCs and tumor cells and DEGs in UGCSCs and normal cells. **(C)** GO function annotation of DEGs in UGCSCs and normal single cells.

Supplementary Table 1 UGIC cell number and target genes

Cell Type	Cell marker	Cell Number
T cell	CD3D; CD2	12207
Epithelial cell	KRT8; KRT18	11461
B cell	MS4A1; CD79A; CD79B	8319
Nature killer cell	CCL3; FCER1G	1534
Fibroblast	PDGFRA; ACTA2	1302
Plasma cell	IGHG3	1477
Cancer Stem cell	EPCAM; TFF2; CD24; ALDH1A1	1586
Mast cell	TPSB2; TPSAB1	679
Endothelial cell	ECSCR; PLVAP	492

Supplementary Table 2 Other Cancer Single-cell data

Cancer Type	Short Name	Sample	Cell	Source
Glioma	GLM	4	3571	GSE84465 (Darmanis et al., 2017)
Glioma	GLM	6	4058	GSE102130 (Filbin et al., 2018)
Melanoma	MELA	5	4645	GSE72056 (Tirosh et al., 2016)
Osteosarcoma	OSTC	11	118326	GSE152048 (Zhou et al., 2020)
Breast Cancer	BC	11	410	GSE75688 (Chung et al., 2017)
Breast Cancer	BC	14	38614	blueprint.lambrechtslab (Qian et al., 2020)
Ovarian Cancer	OVC	10	38953	blueprint.lambrechtslab (Qian et al., 2020)
Stellate Cell Cancer	SCC	10	6341	GSE89567 (Venteicher et al., 2017)

Supplementary Table 3 Gene function annotation

Gene	State	Function
GAST	Up	GAST gene encodes hPG80, which is activated by Wnt signal pathway(Koh and Chen, 2000). hPG80 promotes angiogenesis, and helps tumor proliferation in gastric cancer and colorectal cancer (Giraud et al., 2016).
CXCL8	Up	The CXCL8-CXCR1/2 is a pathway related to the inflammation defense mechanism. Starting from infection and mucosal damage, CXCL8 recruits granulocytes at infection site to eliminate bacteria and inflammatory stimulation. At the same time, the CXCL8-CXCR1/2 pathway activate CXCR2 expression and angiogenesis in endothelial cells of multiple human cancers, which induces CSC invasion and migration (Ha et al., 2017).
BPIFB1	Up	BPIFB1, BPI fold containing family B member 1, regulates infection and chronic inflammation. BPIFB1 is abnormally expressed in nasopharyngeal carcinoma and gastric cancer, indicating that it is significant in tumor development (Li et al., 2020).
REG1A	Up	REG1A, regenerating islet-derived 1α, participate in Wnt/β-catenin triggered signaling pathway in esophageal cancers and colorectal cancers (Sha et al., 2019).
TFF3	Up	TFF3, Trefoil factor 3, is involved in mucosa protection and epithelial cell reconstruction in normal tissues of the digestive system. In gastric cancer, TFF3 activates the PI3K/Akt signaling and accelerates tumorigenesis via the Leptin/ObRb/STAT3 (Inagaki-Ohara et al., 2014; Sun et al., 2014).
PIGR	Up	PIGR is up-regulated in the inflamed intestine and is related to the down-regulation of IL-17 (Kakiuchi et al., 2020).
ZG16B	Up	ZG16B, also known as pancreatic cancer upregulation factor, promotes apoptosis and activates the Wnt/β-catenin pathway in colorectal cancers and promotes cancer progression, which promotes cancer progression (Escudero-Paniagua et al., 2020).
RNASE1	Up	RNASE1 is an important host defense enzyme where it degrades the RNA of viruses and bacteria in inflammatory response. Up-regulated expression of RNASE1 plays an important role in the occurrence and metastasis of gastric cancer (Wang et al., 2006).
CXCL3	Up	CXCL3 is highly expressed in colorectal cancer as a supporting gene of CXCL8 (Ha et al., 2017).

References

Chung, W., Eum, H.H., Lee, H.-O., Lee, K.-M., Lee, H.-B., Kim, K.-T., Ryu, H.S., Kim, S., Lee, J.E., Park, Y.H., 2017. Single-cell RNA-seq enables comprehensive tumour and immune cell profiling in primary breast cancer. Nature communications 8, 1-12. doi:10.1038/ncomms15081

Darmanis, S., Sloan, S.A., Croote, D., Mignardi, M., Chernikova, S., Samghababi, P., Zhang, Y., Neff, N., Kowarsky, M., Caneda, C., 2017. Single-cell RNA-seq analysis of infiltrating neoplastic cells at the migrating front of human glioblastoma. Cell reports 21, 1399-1410. doi:10.1016/j.celrep.2017.10.030

Escudero-Paniagua, B., Bartolomé, R.A., Rodríguez, S., De los Ríos, V., Pintado, L., Jaén, M., Lafarga, M., Fernández-Aceñero, M.J., Casal, J.I., 2020. PAUF/ZG16B promotes colorectal cancer progression through alterations of the mitotic functions and the Wnt/β-catenin pathway. Carcinogenesis 41, 203-213. doi:10.1093/carcin/bgz093

Filbin, M.G., Tirosh, I., Hovestadt, V., Shaw, M.L., Escalante, L.E., Mathewson, N.D., Neftel, C., Frank, N., Pelton, K., Hebert, C.M., 2018. Developmental and oncogenic programs in H3K27M gliomas dissected by single-cell RNA-seq. Science 360, 331-335. doi:10.1126/science.aao4750

Giraud, J., Failla, L.M., Pascussi, J.-M., Lagerqvist, E.L., Ollier, J., Finetti, P., Bertucci, F., Ya, C., Gasmi, I., Bourgaux, J.-F., 2016. Autocrine secretion of Progastrin promotes the survival and self-renewal of colon cancer stem–like cells. Cancer research 76, 3618-3628. doi:10.1158/0008-5472.CAN-15-1497

Ha, H., Debnath, B., Neamati, N., 2017. Role of the CXCL8-CXCR1/2 axis in cancer and inflammatory diseases. Theranostics 7, 1543. doi:10.7150/thno.15625

Inagaki-Ohara, K., Mayuzumi, H., Kato, S., Minokoshi, Y., Otsubo, T., Kawamura, Y., Dohi, T., Matsuzaki, G., Yoshimura, A., 2014. Enhancement of leptin receptor signaling by SOCS3 deficiency induces development of gastric tumors in mice. Oncogene 33, 74-84. doi:10.1038/onc.2012.540

Kakiuchi, N., Yoshida, K., Uchino, M., Kihara, T., Akaki, K., Inoue, Y., Kawada, K., Nagayama, S., Yokoyama, A., Yamamoto, S., 2020. Frequent mutations that converge on the NFKBIZ pathway in ulcerative colitis. Nature 577, 260-265. doi:10.1038/s41586-019-1856-1

Koh, T.J., Chen, D., 2000. Gastrin as a growth factor in the gastrointestinal tract. Regulatory peptides 93, 37-44. doi:10.1016/s0167-0115(00)00176-2

Li, J., Xu, P., Wang, L., Feng, M., Chen, D., Yu, X., Lu, Y., 2020. Molecular biology of BPIFB1 and its advances in disease. Annals of translational medicine 8. doi:10.21037/atm-20-3462

Qian, J., Olbrecht, S., Boeckx, B., Vos, H., Laoui, D., Etlioglu, E., Wauters, E., Pomella, V., Verbandt, S., Busschaert, P., 2020. A pan-cancer blueprint of the heterogeneous tumor microenvironment revealed by single-cell profiling. Cell research 30, 745-762. doi:10.1038/s41422-020-0355-0

Sha, Y.-L., Liu, S., Yan, W.-W., Dong, B., 2019. Wnt/β-catenin signaling as a useful therapeutic target in hepatoblastoma. Bioscience reports 39, BSR20192466. doi:10.1042/BSR20192466

Sun, Z., Liu, H., Yang, Z., Shao, D., Zhang, W., Ren, Y., Sun, B., Lin, J., Xu, M., Nie, S., 2014. Intestinal trefoil factor activates the PI3K/Akt signaling pathway to protect gastric mucosal epithelium

from damage. International journal of oncology 45, 1123-1132. doi:10.3892/ijo.2014.2527

Tirosh, I., Izar, B., Prakadan, S.M., Wadsworth, M.H., Treacy, D., Trombetta, J.J., Rotem, A., Rodman, C., Lian, C., Murphy, G., 2016. Dissecting the multicellular ecosystem of metastatic melanoma by single-cell RNA-seq. Science 352, 189-196. doi:10.1126/science.aad0501

Venteicher, A.S., Tirosh, I., Hebert, C., Yizhak, K., Neftel, C., Filbin, M.G., Hovestadt, V., Escalante, L.E., Shaw, M.L., Rodman, C., 2017. Decoupling genetics, lineages, and microenvironment in IDH-mutant gliomas by single-cell RNA-seq. Science 355. doi:10.1126/science.aai8478

Wang, L., Zhu, J.-S., Song, M.-Q., Chen, G.-Q., Chen, J.-L., 2006. Comparison of gene expression profiles between primary tumor and metastatic lesions in gastric cancer patients using laser microdissection and cDNA microarray. World journal of gastroenterology: WJG 12, 6949. doi:10.3748/wjg.v12.i43.6949

Zhou, Y., Yang, D., Yang, Q., Lv, X., Huang, W., Zhou, Z., Wang, Y., Zhang, Z., Yuan, T., Ding, X., 2020. Single-cell RNA landscape of intratumoral heterogeneity and immunosuppressive microenvironment in advanced osteosarcoma. Nature communications 11, 1-17. doi:10.1038/s41467-020-20059-6
